# Parental experiences of perinatal loss, with a focus on hospice provision: A thematic analysis

**DOI:** 10.1017/S1478951525101223

**Published:** 2026-01-08

**Authors:** Rhiannon Latham, Katrina Williams, Keeley Guest, Fauzia Paize, Robyn Lotto

**Affiliations:** 1 Faculty of Health, Innovation, Technology and Science, LJMU, Liverpool, UK; 2 Liverpool Women’s Hospital NHS Foundation Trust, Claire House Children’s Hospice, Liverpool, UK; 3 Liverpool Women’s Hospital NHS Foundation Trust, Liverpool, UK

**Keywords:** Perinatal palliative care, qualitative, parent voice

## Abstract

**Objective:**

Perinatal palliative care (PPC) offers holistic support to families of babies with life-limiting conditions, addressing emotional, psychological, and practical needs alongside ensuring dignity for the baby. While there is growing evidence to support its benefits, there remains inconsistent service provision, limited integration with maternity care, and regional disparities. This study explores parental experiences with perinatal hospice services to inform future care models.

**Methods:**

The study was undertaken in the Northwest of England. Fourteen semi-structured interviews were undertaken with 17 parents (three joint interviews) who had experienced perinatal loss and had engaged with PPC services. Semi-structured interviews were used to gather insights into their perceptions of care they received, focusing on issues such as communication, the timing of referrals, and the emotional and practical support provided. Data was analyzed using a thematic analysis approach.

**Ethical approval:**

The obtained REC reference: 22/YH/0028 Results Five key themes were identified: the significance of language used by healthcare professionals when discussing the baby’s condition; the importance of timely introduction to hospice care; recognition that grief is a personal and evolving process; the role of shared experiences in building relationships; and the importance of creating lasting memories.

**Significance of results:**

Findings highlight the importance of improving healthcare professionals’ communication skills and integrating multidisciplinary palliative care services early in the care pathway. Parents expressed gratitude for the hospice support, particularly the opportunity to spend quality time with their baby and make lasting memories. However, a more consistent perinatal hospice care provision across the UK is needed.

## Introduction

Perinatal palliative care (PPC) is a specialized form of support provided to families of infants diagnosed with a life-limiting or life-threatening condition (Dombrecht et al. 2023). It is a comprehensive care approach aimed at enhancing the quality of life and ensuring comfort for a baby during the perinatal period (ACOG 2019), with families accepted onto the PPC pathway from point of suspicion or diagnosis of a significant anomaly (Wilkinson et al. 2025). It focuses on improving quality of life and maximizing the quality of the time families spend together by addressing physical comfort, emotional, social, and spiritual needs. It promotes a multidisciplinary approach with shared decision making with parents, compassionate communication and coordinated care helping families create meaningful experiences with their baby whistle preparing for the potential for ongoing life, end of life care, death, and bereavement support (Together for Short Lives 2017). The perinatal period is typically defined as spanning from the 22nd week of gestation to 7 days after birth (WHO 2016), but the timeframe for PPC is more flexible, with some services extending support up to 18 months postnatally. Reviews of PPC highlight variation in duration and models of care (Dombrecht et al. 2023). While some services focus on the antenatal and immediate postnatal period, others continue beyond infancy to address ongoing medical, emotional, and bereavement needs (Bertaud et al. 2023; Korzeniewska-Eksterowicz et al. 2025). This variation reflects differing service structures, referral pathways, and definitions of “perinatal” care, underscoring the need for flexible, family-centered approaches responsive to individual circumstances.

Despite advances in perinatal care, infant mortality rates of approximately 5.8 deaths per 1,000 live births continue to be reported in high-income countries, with prematurity and chromosomal/congenital anomalies listed as the primary contributing factors (Callaghan et al. 2017; Mathews and Driscoll 2017). While advances in perinatal care have improved neonatal outcomes, particularly for extremely preterm infants, only 20% of infants born at 22–24 weeks gestation survive without any neurodevelopmental impairment (Younge et al. 2017), highlighting the ongoing challenge of unfavorable diagnosis and poor prognoses. In response to these challenges, specialized PPC has been steadily gaining recognition as a crucial support system for families navigating these difficult circumstances, playing a key role in addressing both the medical care of the baby and emotional needs of their families (Gomes Guimarães et al. 2018). However, in the United Kingdom (UK), the expansion of dedicated perinatal hospices and services remains limited and inconsistent, with gaps in the integration of maternity and palliative care services leading to regional variations in accessibility (Mitchell et al. 2021; Perinatal Hospice and Palliative Care 2021). While this study is situated in Northwest England, where hospice-based PPC provision is relatively established, regional variation persists across the UK. Some regions have integrated PPC within neonatal networks, whereas others have limited or no access to dedicated hospice services (Mitchell et al. 2021; Tatterton et al. 2023). Overall, PPC continues to be delivered primarily within hospital maternity and neonatal units, with only limited specialist provision available through children’s hospices nationwide. (Tatterton et al. 2023). Despite the growing evidence of positive outcomes from engagement with hospice care (Boan Pion et al. 2021; Mitchell et al. 2021), provision and uptake remains low (Mendizabal-Espinosa and Price 2021). Barriers to referral include the common misconception that “palliative care” is solely associated with end-of-life care, as well as the wide range of conditions eligible for perinatal palliative support, from extreme prematurity to complex congenital anomalies. This can create uncertainty around when and for whom such care should be offered (Benini et al. 2020). This is further complicated by diagnostic uncertainty, which often results in hesitation among healthcare professionals (HCPs) to initiate discussions, consequently delaying critical support for families (Wool et al. 2016).

Further systemic barriers, including the absence of standardized referral pathways and limited interdisciplinary collaboration, hinder the seamless integration of PPC into routine clinical practice (Dombrecht et al. 2023). While the need for timely referrals to specialist services is well recognized, with care pathways designed to foster continuity of care, relationship-building with professionals, and shared decision-making (Together for Short Lives 2017) the knowledge, experience, and confidence of HCPs continue to influence the options presented to parents. Preconceived notions and varying levels of familiarity with PPC contribute to inconsistencies in referral practices, ultimately leading to disparities in the experiences and care accessed by families (Wool C 2013; Peng et al. 2018).

This paper seeks to explore the experiences of parents who have navigated a bereavement of their baby with support of perinatal hospice services, drawing insights from their personal narratives.

## Methods

Qualitative data was collected via semi-structured interviews with parents who had experienced a perinatal death, to gain a deeper understanding of the complexities involved in caring for this cohort. All parents interviewed had received care from a palliative care team, as described in Appendix 1.

### Sample and recruitment

A total of 24 parents (12 couples) were approached. All had experienced a perinatal death over a 2-year period between 2021 and 2023 and had received care from a hospice PPC team. Parents who were identified as being vulnerable or experiencing complicated grief reactions were excluded by clinical staff prior to invitations being sent out. Purposive sampling was used to ensure representation of a broad spectrum of experience, including those who had been advised of problems relating to the pregnancy antenatally, those who had received a postnatal or postmortem diagnosis, and those who had delivered prematurely, resulting in the death of the baby. In addition, a sample of parents were included whose baby had died in utero, in hospital, or had been transferred to a children’s hospice for end-of-life care. A total of 14 interviews were conducted, involving 17 parents and reflecting 12 pregnancies. In 2 cases, both parents participated separately in individual interviews, while 3 couples chose to be interviewed together. The remaining interviews were undertaken with either the mother or the father, depending on participant preference. Overall, the sample included 6 fathers and 11 mothers. To maintain participant anonymity, detailed demographic information has not been included. However, a brief description of the sample can be viewed in Tables 1 and 2.

**Table 1.** Sample descriptors

		Sample size
Cause of death	Antenatal diagnosis of congenital anomaly	9
	Postnatal diagnosis of a congenital anomaly	2
	Extreme prematurity	4
Place of death	In hospital death	6
	In hospice death	6
	In utero death	3

^a^ Sample includes a number of multiple pregnancies, plus early fetal loss in one multiple pregnancy. The total numbers are therefore greater than the number of couples.

**Table 2.** Interview descriptors

	Interview/participant number
Mother only	2, 3, 4, 8, 10, 11, 13, 14
Father only	5, 9, 12
Joint interview	1, 6, 7

Consistent patterns were identified within the first 5 interviews, with later interviews largely reinforcing previously observed findings rather than introducing novel perspectives. However, recruitment continued to ensure inclusion of both parents and differing care pathways. Ethical approval for the study was granted by HRA and Health and Care Research Wales REC reference: 22/YH/0028.

### Data collection

The interview schedule was devised by a small patient and public involvement group and trialed before data collection commenced. Semi-structured interviews were conducted either face to face (*n* = 12) or online (*n* = 2) by RL. Data were collected between July 2022 and July 2023. The interviews averaged 1 hour 20 minutes in length (range 55 minutes to 2 hours 10 minutes). A reflective diary was maintained by RL after each interview to provide further context.

### Data analysis

Opportunities for participants to verify the findings were provided throughout the analysis process. All interviews were recorded digitally, anonymized, and transcribed verbatim. Participants were also invited to review their transcripts. However, only 2 participants requested to do so. No changes were made to the original transcript following review.

Data analysis employed a reflexive thematic analysis approach (Braun and Clarke 2022). Each transcript was read by at least 2 authors (RL, RSL, KG) to facilitate data familiarization. The authors (RSL, KG) then independently coded and generated initial themes. The analysis and evolving themes were discussed with the other authors (RL, FP, KW) to help develop analytical insights. Participants universally praised the support provided by the hospice, with many highlighting the compassion and professionalism of the clinical teams from the time of referral. For many, the hospice became a vital source of comfort, providing reassurance during an immensely challenging time. Following the death of their baby, activities such as fundraising and hospice promotion offered participants meaningful ways to remain connected, fostering a sense of purpose and ensuring their baby’s memory lived on.

Reflexive thematic analysis was selected for its flexibility and suitability in exploring subjective experiences, acknowledging the active role of the researcher in meaning-making (Braun and Clarke 2022). The 6-phase approach guided the process: (1) data familiarization, (2) generating initial codes, (3) searching for themes, (4) reviewing themes, (5) defining and naming themes, and (6) producing the report. This iterative framework enabled reflexivity, allowing the authors to continually revisit interpretations and ensure that themes evolved through collective discussion and analytic depth.

## Findings

**Figure 1.**
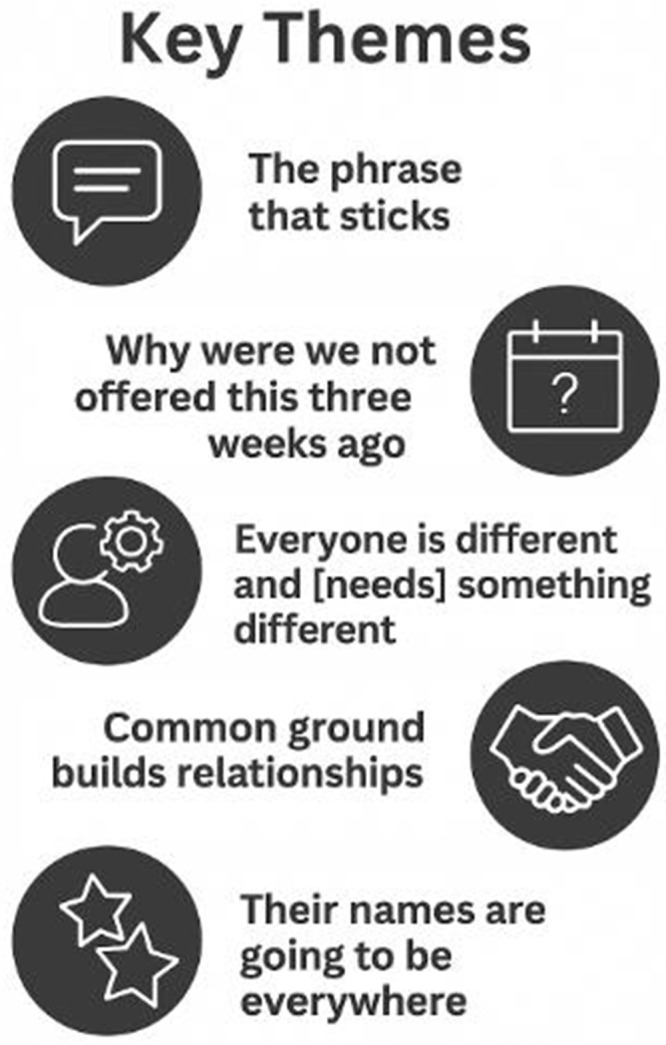
Key themes.

Five key themes were identified (Fig. 1) relating to different points within their journey: (I) “the phrase that sticks”; (II) “why were we not offered this three weeks ago?” highlight the importance of HCP’s use of language and the timing of this communication when discussing the baby’s ill health, treatment, and their introduction to the concept of PPC; (III) “everyone is different and [needs] something different” acknowledges that grief is a highly individual and non-linear process, with parents’ subsequent support needs varying significantly; (IV) “common ground builds relationships” emphasizes the connection or distance created in response to grief; and (V) “their names are going to be everywhere” stresses the importance of keeping their child’s memory alive. Exemplar quotes are provided in Table 3.

**Table 3.** Participant quotes arranged by theme

Theme	Exemplar Quote	Quote Number
***Theme 1: “The phrase that sticks”***	*“It was these mixtures of language, and especially at that time we were so sensitive to language.” (Interview 4)*	Q1.1
	*“The phrase that one of the doctors used to us which really sticks with me. And it was, this baby isn’t viable… I imagine she wanted to be more compassionate than she came across but that’s the way it was used. And the impact on us… it sticks with me today that phrase.” (Interview 11)*	Q1.2
	*“And then eventually… that was when I did fight, I had to put in a complaint to get an appointment with her in the first place, it was just couldn’t get any support from her.” (Interview 2)*	Q1:3
	*“I think the doctors may have assumed that the previous consultant had told us outcomes and things and finds that haven’t been communicated with us.” (Interview 9)*	Q1.4
	*“In the meantime, we went out to a private foetal medicine consultant… we just struggled with not having a diagnosis and everyone telling us they didn’t know what it was, and they’d never seen this before.” (Interview 7 – father)*	Q1.5
	*“I’ve said, “Oh, I knew it, we’ve been to see a private consultant and that’s one of the things I asked him” and then her whole demeanor just changed; “You’re welcome to take your care elsewhere.” (Interview 1 – father)*	Q1.6
	*“There was one occasion when [Consultant] spent it felt like all night with us. There didn’t feel like any rush and they answered our questions, it made such a difference. We felt like we actually understood what was happening and could trust them.” (Interview 10)*	Q1.7
	*The doctor was careful… he said, “Hopefully everything is going to turn out fine, but they will support you either way.”” (Interview 8)*	Q1.8
Theme 2*: “Why were we not offered this three weeks ago?”*	*“He said look, they do look after people who are palliative, young people who are palliative, but they also look after people who, you know, you just, your baby is very poorly and you need the extra support and hopefully everything is going to turn out fine, but they will support you either way regardless… so he explained it really well to me and that’s why I accessed [hospice].” (*Interview *2)*	Q2:1
	*“Then the midwife just said, “You can see [hospice]. Someone from [hospice] can come. [Husband] was beside himself, you know, “It’s a hospice, our baby’s going to die. What do we do?” (Interview* 3)	Q2:2
	*“I actually said to her, “why were we not offered this three weeks ago?” and you just get told, “Well, parents don’t take kindly to it,” I’m like, “Well your paternalistic viewpoints are taking that choice away from people, it’s not your decision to make.”* (Interview 7 – Mother).	Q2:3
	*“They [hospice staff] came over quite frequently… I suppose at first when you hear that they’re from a hospice you kind of think, you know, but I think they probably visit more parents with very, very premature babies, but I do think they visit nearly all of them there. When we’d had the talk about palliative care, and [hospice staff] was one of the first people that I rang. You know, her and [hospice staff] knew [baby], so they were already familiar with us.”* (Interview 8)	Q2:4
	*We just thought that [hospice] were there just to support us. Just because of the situation. I never thought about palliative care, or that he was going to pass away.” (Interview 14)*	Q2:5
	*“It felt like [Claire House] was the only time I’d had any support. Even after finding out about the heart condition, my midwife didn’t even say… ‘here’s what you can do.’ She was still awful.” (Interview 2)*	Q2:6
	*“When they say where they’re from… people are like, ‘Oh my god, is my baby dying?’ But we never thought that… we just thought they were there to support us.” (Interview 1 – Mother)*	Q2:7
*Theme 3: “Everyone’s different and [needs] something different”*	*Talking to wife: “You really get a lot of help from it, but I find it’s a lot of the same conversations if you know what I mean. Like people are dealing with the same issues. And it’s good for them, they can get it off their chest. But, for some of us a bit introverted like me, I don’t really contribute that much.” (Interview 6 – Father)*	Q3.1
	*“Because when I had counselling, I didn’t feel like I needed it. But, I said, I probably will do in the future.” (Interview 1)*	Q3.2
	*“Yeah, I am sure many dads feel that, feeling a bit spare, I think, sometimes. Like I say my main and only focus was [wife] and [baby], so yeah, it was very much not in my thinking about how I am doing, that sort of thing.” (Interview 5)*	Q3.3
	*“You do feel really it’s a bit chauvinistic, but you do feel responsible for your wife as a husband. I know you shouldn’t, you know these days, but that’s just the way we feel. And to have to see what she’s managing well, is a big relief.” (Interview 12)*	Q3.4
	*“We were talking about there’s pros and cons, because we never actually got any time to grieve alone, because the kids were off school for a couple of weeks afterwards… I was careful how I said it, because I was conscious they didn’t have other children and they would have loved that distraction.” (Interview 2)*	Q3.5
	*“Most of them have still got one child left, which is my only criticism. Because you are mixed with people who have still got children around.” (Interview 1 – mother)*	Q3.6
**Theme 4: *“Common ground builds relationships”***	“*It just becomes a community. It’s like a family that you are part of, you don’t want to be in it, but you are.” (Interview 11)*	Q4.1
	*“Relationships have also struggled in our family, people who can’t quite understand what we’re going through and the fact that time doesn’t massively heal things.” (Interview 8)*	Q4.2
	*“The bereaved parents in the room, it’s like speaking a language that you’re familiar with and of course if you’re outside of that circle, whether it be family or friends, they’re very sympathetic but they can’t truly understand.” (Interview 14)*	Q4.3
	*“So, it’s just far better for us having these conversations, with likeminded people here than trying to have this, expect friends and families to come up with the goods.” (Interview 9)*	Q4.4
	*“So they never held him… Because it’s strange because we were in the hospital with him. And we shared pictures and things. It’s like it sort of didn’t happen.” (Interview 1)*	Q4.5
	*“Initially family were supportive. It’s just sometimes… Family [dynamics] can be a bit negative sometimes. And thinking that we should be over it by now, and stuff.” (Interview 7 – mother)*	Q4.6
	*“My cousin blurts out last week that she thought we should be getting over it. So, yeah, it’s kind of… it does set me back a bit.” (Interview 13)*	Q4.7
	*“It’s nice to come once a month… because we are like friends now, aren’t we? And friends who don’t say things [like family sometimes do].” (Interview 8)*	Q4.8
	*“It’s people who understand. You don’t need to say anything to them half the time because clearly, they already know.” (Interview 10)*	Q4.9
	*“People said, ‘You should be over it by now.’ It’s not supportive; it’s judgmental and sets you back.” (Interview 11)*	Q4.10
**Theme 5: *“Their names are going to be everywhere”***	*“They make it lovely, they do painting with him, they do everything with him, family come to visit and just like play with them and they have a pool and everything. So he thought it was like Butlins.” (Interview 4)*	Q5.1
	*“I feel like, he felt in ways, although he passed away, he felt more mine. Because I could pick him up whenever I wanted to. And I could sit and cuddle him, there wasn’t the tubes, and the struggling to move. It was a proper cuddle.” (Interview 6 – mother)*	Q5.2
	*“Doing the abseiling. I’m collecting all my money in this week. I’ve raised about £550. So it’s a lot it’s just to give the children a day to make memories, a day out, just to give their parents something to keep.” (Interview 10)*	Q5.3
	*“I love it when people say their names, I love it when people talk about them but I understand some people find it hard but it’s when people just don’t acknowledge.” (Interview 3)*	Q5.4
	*“We take the [bears] with us when we go away somewhere… That they both had, didn’t they? After they had passed away.” (Interview 6 – mother)*	Q5.5
	*“We’re going to [names town] on Sunday. Because we have put their names on the side of… the wheelhouse of the lifeboat.” (Interview 1 – mother)*	Q5:6
	*“We had posters on the wall about 30 of us… a sponsored walk. And some parents from here… We raised over £4,000 in just that.” (Interview 5)*	Q5:7
	*“We’ve got stars named after them. Their names are going to be everywhere.” (Interview 7 – mother)*	Q5:8

### Theme 1: “the phrase that sticks”

Participants emphasized the profound impact of the language used by HCPs when discussing their baby’s condition and treatment plan. Insensitive or ambiguous terms, often perceived as overly clinical or medicalized, heightened feelings of uncertainty and distress (Q1.1, Q1.2). Conversely, clear, compassionate communication significantly alleviated anxiety and fostered trust in the care team (Q1.7, Q1.8).

In some cases, participants expressed frustration when they received conflicting or inadequate information or felt their concerns were dismissed by HCPs (Q1.3, Q1.4). This lack of clarity often prompted parents to seek additional information from external sources, including the internet, social media, or private healthcare providers (Q1.5). While these efforts sometimes enhanced understanding, they also risked undermining the parent-HCP relationship (Q1.6).

### Theme 2: “why were we not offered this three weeks ago?”

The timing and framing of communication about hospice services were pivotal in shaping parents’ experiences. Participants noted that language which normalized palliative care and emphasized its supportive role helped to reduce fear and stigma, enabling them to engage more openly (Q2.1). Many expressed a preference for earlier discussions about hospice care providing referrals were accompanied by clear explanations of the available services and their purpose (Q2.3, Q2.4).

Parents’ initial reactions to hospice referrals were often shaped by their pre-existing perceptions of hospice care (Q2.7). Those with inaccurate or incomplete understandings associated it with a sense of finality, resulting in more negative responses (Q2.2). Additionally, some participants felt that HCPs delayed referrals based on assumptions about how parents might react, further complicating their access to support (Q2.3). However, gentle introductions to hospice staff, such as informal ward visits, helped build familiarity and eased the transition into hospice care (Q2.4, Q2.5).

### Theme 3: “everyone’s different and [needs] something different”

Participants consistently highlighted the individual nature of grief, acknowledging that support needs varied greatly and evolved over time (Q3.1, Q3.2). Fathers often prioritized their partner’s wellbeing, as mothers faced both the physical and emotional challenges of pregnancy, birth, and loss. As a result, fathers were initially less likely to directly engage with hospice services but indirectly benefited from the support their partners received (Q3.3, Q3.4).

Participants also reflected on the varying experiences and needs of other bereaved parents. These differences sometimes created challenges in connecting with others whose circumstances differed, such as parents with living children finding it difficult to relate to those without, and vice versa (Q3.5, Q3.6).

### Theme 4: “common ground builds relationships”

Shared experiences of bereavement through baby loss created strong bonds among participants, with the hospice providing a safe and understanding environment where they could openly share their emotions and feel a sense of belonging (Q4.1, Q4.3, Q4.4). However, participants also described feelings of isolation stemming from a lack of understanding among family, friends, and colleagues (Q4.2, Q4.5, Q4.10). This disconnect arose from others’ inability to fully empathize with the complexity of their grief.

Participants recognized that while family and friends were generally well-intentioned in their efforts to provide support, they often lacked the lived experience necessary to offer the same level of understanding as those who had undergone similar losses (Q4.6, Q4.7). Many participants reported that, despite these supportive efforts, loved ones occasionally struggled to articulate appropriate responses, sometimes making comments that were perceived as dismissive or unintentionally hurtful (Q4.7). This discrepancy in understanding underscored the distinct value of peer support networks and bereavement groups, which provided a more empathetic and validating space for emotional expression and coping (Q4.8, Q4.9).

### Theme 5: “their names are going to be everywhere” (q5.8)

Preserving their baby’s memory was a central priority for participants. Many emphasized the value of spending time with their baby in the calm, non-medicalized environment of the hospice, which helped strengthen their bond as a family (Q5.1, Q5.2).

Keepsakes such as hand and footprint jewelry were deeply cherished, with participants describing how these tangible items provided comfort and connection (Q5.5. Q5.6), with many holding these items during the process of being interviewed. Acts of remembrance, such as fundraising and other memorial activities, allowed parents to honor their baby while contributing to the hospice’s ongoing work (Q5.3, Q5.7). Participants also expressed a strong desire for others, including family and friends, to recognize their baby’s personhood and remember them as an integral part of the family’s story (Q5.8). Conversely, avoidance of acknowledging the baby was perceived as hurtful (Q5.4).

## Discussion

This study explored the experiences of 17 parents who engaged with PPCPPC in Northwest England. The findings underscore the importance of timely, empathetic communication, personalized support, and opportunities for shared connection and remembrance.

A consistent theme relates to the way in which PPC was framed and introduced. Many participants initially held limited or inaccurate understandings of PPC, often equating it solely with death or the discontinuation of medical care. This perception, rooted in societal discomfort and clinical ambiguity around the term “palliative,” contributed to initial resistance and anxiety (Saad et al. 2022). Framing PPC as an extension of supportive antenatal care rather than solely an end-of-life intervention has been associated with improved parental engagement and service accessibility (Cote-Arsenault and Denney-Koelsch 2011).

Similarly, clinician hesitancy, uncertainty around prognosis, and concerns about overwhelming parents often contribute to delayed introductions of PPC (Wool and Catlin 2019; Beltran and Hamel 2021). However, early, sensitively delivered discussions, tailored to parental readiness, can improve engagement and decision-making, ensuring families feel informed and supported from the point of diagnosis (Balaguer et al. 2012; Saad et al. 2022).

Delayed referrals have also been linked to missed opportunities for holistic care planning, limiting access to psychological, spiritual, and practical support when it may be most beneficial (Beltran and Hamel 2021). These experiences reflect broader challenges in PPC implementation, where clinician confidence and institutional norms vary widely risking inconsistent referral practices (Beltran and Hamel 2021; Hardicre et al. 2021). Addressing these barriers requires a shift toward proactive communication strategies and structured referral pathways, integrating PPC discussions into routine antenatal care and prioritizing shared decision-making and parental autonomy (Silveira et al. 2023)

To address these gaps, systemic investment in workforce development is essential. Training programs should emphasize empathetic communication, trauma-informed care, and managing prognostic uncertainty (Beltran and Hamel 2021). This aligns with global best practices, which emphasize the importance of building parental trust, fostering early engagement, and enhancing preparedness for families navigating complex pregnancies (Wool and Catlin 2019; Saad et al. 2022). Additionally, incorporating the question “would I be surprised if this baby was to die in the perinatal period?” could help HCPs identify families who may benefit and make appropriate, early referrals (Asenjo et al. 2025).

Creating standardized referral protocols, especially within antenatal care, can ensure that PPC is offered consistently and equitably, regardless of individual clinicians’ comfort levels. Integration of PPC into routine maternity services through shared care planning and multidisciplinary collaboration would further support early, seamless engagement with families (Tatterton et al. 2023). Several participants found that informal introductions to hospice teams, such as ward visits, helped normalize PPC and build trust. These gentle strategies allowed parents to engage at their own pace, reducing anxiety and stigma. International models also support phased introductions and peer-support initiatives to build confidence in PPC engagement (Hein et al. 2022).

The study also highlighted the emotional and social value of peer support and memory-making. Many parents formed deep emotional bonds through shared experiences of baby loss, particularly within the hospice setting. In contrast, participants described feelings of isolation from family, friends, and colleagues who lacked the lived experience to fully understand their grief. Peer networks offered a space for validation, empathy, and normalization of grief, helping parents feel less alone (Badenhorst and Hughes 2007; Zhuang et al. 2023). Involving the wider multi-generational family within hospice settings has been shown to strengthen support networks for bereaved parents, helping to challenge misconceptions about grief and encourage the continued presence of the baby in family traditions (Hein et al. 2022; Jackson et al. 2023). Increasing evidence highlights the value of family-inclusive grief support, particularly in cultures where perinatal loss is stigmatized or rarely acknowledged (Fernández-Sola et al. 2020). Global models of bereavement care increasingly recognize the significance of meaning-making practices, with studies demonstrating that rituals and community-driven remembrance events enhance emotional adjustment and reduce prolonged grief symptoms (Nyatanga 2020).

Preserving the baby’s memory was central to participants’ coping processes. Time spent in a calm, non-medical environment and access to memory-making opportunities such as keepsakes, photography, and symbolic rituals, reinforced their sense of parenting and connection. Acts of remembrance, including fundraising and advocacy, provided parents with a continued sense of purpose and identity. These practices align with bereavement models that emphasize meaning-making and continued bonds as vital for emotional recovery (Anolak et al. 2019; Hart et al. 2022).

Previous research has demonstrated that parental experiences of perinatal loss are influenced by a range of demographic and clinical factors, including ethnicity, socioeconomic context, gestational age, and cause of death (Zhang et al. 2024). Cultural norms and family expectations have been shown to shape how grief is expressed and supported (Fernández-Sola et al. 2020), while the timing and circumstances of loss can influence emotional adjustment and opportunities for memory-making (Jackson et al. 2023; Zhuang et al. 2023). These factors are likely to hold relevance for the current study cohort. However, further investigation is needed to understand how such demographic and clinical variations may impact experiences of and engagement with PPC across diverse populations.

### Recommendations

The study builds on previous research findings, adding to the body of knowledge and acknowledgement that further training and education is required for HCPs that work in neonatal and maternity services and are responsible for delivering bad news and introducing hospice services.

Efforts must be directed toward improving the integration of PPC within maternity and neonatal care pathways. Currently, inconsistent service provision and gaps in accessibility reflect a need for systemic changes that prioritize equitable access to specialized PPC services across regions (Mitchell et al. 2021). Strengthening collaboration between hospital-based maternity services and hospice care providers is critical to creating a seamless care experience for families. Additionally, enhancing HCPs’ knowledge and confidence in discussing PPC options through targeted training can reduce disparities in referral practices and ensure that families receive timely support. Implementing structured care pathways, such as those recommended by Together for Short Lives, could promote consistency, continuity of care, and shared decision-making, ultimately improving outcomes for bereaved families (Together for Short Lives 2017). These measures, combined with ongoing evaluation and feedback, would help address the variability in PPC delivery and better meet the emotional, practical, and psychological needs of families during perinatal loss.

### Strengths and limitations

The study’s inclusion of both mothers and fathers provides a comprehensive understanding of parental experiences, capturing diverse perspectives that are often underrepresented in PPC research. However, couples recruited were all “mother/father” and therefore the needs of same sex couples have not been explored. Reflexive thematic analysis of the transcripts was employed by two authors, one of whom has clinical experience in palliative care (KG) and one of whom was novel to the research area (RSL) to help reduce subjective bias.

## Conclusion

This qualitative study explored the experiences of parents navigating perinatal loss with the support of PPC services in the Northwest of England. The findings underscore the critical role of timely, sensitive communication, personalized support, and opportunities for memory-making in facilitating parents’ journeys through grief. Parents highlighted the profound impact of language used by HCPs, with clear, empathetic, consistent communication fostering trust and reducing distress. Delays in PPC referrals were a common concern, with participants expressing a preference for earlier engagement that could have allowed more meaningful time with their baby and smoother grief processing.

The study also emphasized the non-linear nature of grief and the need for flexible, individualized support, particularly for fathers, whose experiences are often under-addressed. It is unclear whether the shared experiences of baby loss within the hospice setting created valuable connections, while acts of remembrance and the opportunity to honor their baby provided parents with comfort and purpose.

To enhance the delivery of PPC, healthcare systems must prioritize training for professionals in empathetic communication, improve integration between maternity and palliative care services, and normalize discussions about PPC early in the care pathway. Expanding access to peer support and formalizing opportunities for families to engage in memory-making could further support bereaved parents. By addressing these key areas, PPC services can better meet the emotional, psychological, and practical needs of families, ensuring compassionate care during an immensely difficult and emotionally complex time.

Collectively, these findings offer valuable insights for enhancing the equity and cultural sensitivity of PPC and can inform the development of national strategies aimed at standardizing referral pathways and strengthening support for bereaved families.

## Appendix 1

The Children’s hospice intervention in this study involved early referrals, support at clinic appointments and scans, emotional support – someone for families to contact between appointments by phone, video call or face to face, separate antenatal classes run by a midwife, parallel birth planning – a concept where information is shared and documented about “hoping for the best” but have a plan “just in case of the worst” focusing on what would be important to a family and capturing wishes if time together as a family was going to be short whilst also incorporating some control into a birth plan with elements of a typical birth plan such as pain relief and positioning for birth. Antenatal memory making is encouraged with free bump photography and heartbeat recordings and there is support for the whole family with sibling support and complimentary therapies such as reflexology and reiki for both parents. Also support on the neonatal unit and in bereavement care if needed. For children who get home a baby drop in group is also available to families for peer support. Further information can be found at https://www.clairehouse.org.uk/.
